# The computation of relative numerosity, size and density

**DOI:** 10.1016/j.visres.2014.12.022

**Published:** 2016-07

**Authors:** Sabine Raphael, Michael J. Morgan

**Affiliations:** a Max-Planck Institute for Neurological Research, Gleueler Strasse 50, Köln, Germany; b Newcastle University, Institute of Neuroscience, Framlington Place, Newcastle upon Tyne NE2 4HH, UK; c City University London, Applied Vision Research Centre, Northampton Square, London EC1V 0HB, UK

**Keywords:** Psychophysics, Texture, Density, Size, Numerosity

## Abstract

To investigate the mechanisms for the perception of relative numerosity, we used two-interval forced-choice (temporal 2AFC) to measure thresholds for area, density and numerosity differences between dot textures, and a 2 × 2 FC task to measure the ability of observers to distinguish changes in area from changes in density. To prevent the use of a one-dimensional size signal we used textures in which dots were scattered within irregular polygonal areas. Numerosity thresholds were similar in the area and density-varying conditions, consistent with a single numerosity mechanism. Thresholds for area and density discriminations were raised when number was held constant, consistent with numerosity thresholds being lower than those for size and density. Also, area thresholds for polygonal outlines were increased when no dots were present in the outline. However, a single numerosity mechanism cannot account for all the data, because we find that observers in randomly-interleaved size-varying and density-varying conditions are also able to discriminate between changes in size and density with a precision predicted from independently-noisy size and density channels that have similar noise to that in the putative numerosity channel. A complication, previously noted with circular shapes, is that denser textures tend to be confused with larger textures, and vice versa. This could explain why thresholds rise when density and size changes are in opposition, in the constant-number case. These findings taken together do not rule out an independent numerosity mechanism, but they are equally compatible with a flexible computation of numerosity from size and density cues.

## Highlights

• Observers could accurately discriminate dot number in irregularly shaped arrays.
• Accuracy was less than that previously reported for regular shaped arrays.
• Observers could discriminate changes of dot density from changes in array size.
• Results are consistent with a computation of number from size and density.

## 1 Introduction

**Fig. 1:**
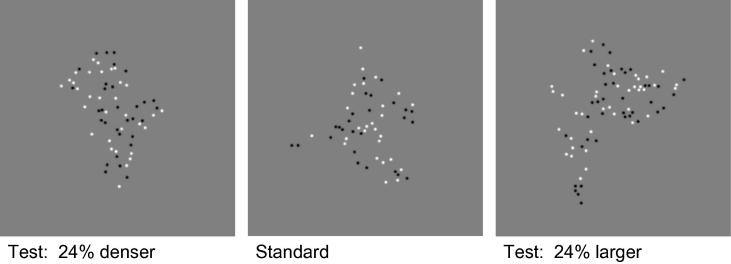
Example stimuli. Left: Test stimulus of the same area as the standard and greater density. Center: Standard stimulus containing 64 dots within the standard area. Right: Test stimulus with larger area than the standard but the same density. The shapes were generated by an algorithm that randomly varied the position and number of vertices in the polygon while keeping area constant.

Relative numerosity discrimination has been studied experimentally in adults (Burr and Ross, 2008, Durgin, 1995, Durgin, 2008, Ross and Burr, 2010) infants (Xu & Spelke, 2000), and non-human species (Brannon et al., 2001, Gallistel, 1989, Leslie et al., 2008), using psychophysics (Barlow, 1978), fMRI (Harvey et al., 2013, Piazza et al., 2007), and single unit physiology (Nieder, 2005). It has been suggested that there is a ‘visual sense of number’ (Burr & Ross, 2008) and that ‘Vision senses number directly’ (Ross & Burr, 2010) for large numbers of tokens. Here we attempt to discover whether there is indeed a mechanism for numerosity separate from density and size of textures. A common-used strategy for measuring relative numerosity thresholds is to scatter the tokens within a confined area, such as a circle (Burr and Ross, 2008, Durgin, 1995, Raphael et al., 2013). In these circumstances, changing the number of tokens must change either the area of the pattern or the density of items. Weber fractions for numerosity are lower when the numerosity change is accompanied by a change in area (Raphael, Dillenburger, & Morgan, 2013), in agreement with other studies showing that a high-precision, one-dimensional mechanism is responsible for area discrimination of circles (Morgan, 2005, Nachmias, 2011). Therefore, experiments with circular textures may overestimate the accuracy of true numerosity discrimination. Randomly interleaving size-varying and density-varying trials (Burr and Ross, 2008, Raphael et al., 2013) does not solve this problem, since observers may use whichever of the two independently noisy signals, size or density, is larger on a particular trial (Raphael, Dillenburger, & Morgan, 2013). For these reasons, we thought it desirable to repeat the experiment of Burr and Ross (2008) using stimuli with non-circular polygonal outlines (Fig. 1). We compared four conditions: (1) density-varying trials alone (2) area varying trials alone (3) interleaved area-density trials where the observers made a numerosity discrimination and (4) which is the same as condition 3, but in addition observers had to decide whether the difference was in area or in density. We expected area thresholds for random polygons to be higher than those for circles, and the first question was whether this would also raise thresholds for numerosity. In additional conditions subjects discriminated changes in density or changes in size when numerosity was constant.

In signal-detection models of the data, we asked whether independently noisy area and density channels were sufficient to account for the data, or whether a separate numerosity mechanism is required. We addressed this question by comparing two-channel vs three channel fits to the combined data in all conditions.

## 2 Methods

### 2.1 Stimuli and procedure

Examples of the stimuli are shown in Fig. 1. Stimuli were presented on the LCD display of a MacBookPro laptop computer with screen dimensions 33 × 20.7 cm (1440 × 900 pixels) viewed at 0.57 m so that 1 pixel subtended a visual angle of 1.25 arcmin. The background screen luminance was 50 cd/m^2^. Stimulus presentation was controlled by MATLAB and the PTB3 version of the Psychtoolbox. On each trial subjects saw consecutively two stimuli, which they were required to compare for number, density or size.

Each stimulus contained a number of fuzzy dots with a diameter of 10 arcmin and a Gaussian envelope with a space constant of 2.5 arcmin. Each dot was randomly assigned a negative (black, 0.4 cd/m^2^) or positive contrast (white, 300 cd/m^2^). The dots were randomly positioned within notional polygons without overlap. The irregular polygon shapes were generated by an algorithm that pseudo-randomly varied the position and number of vertices of each polygon in any trial. In all conditions the standard stimulus contained 64 dots within the standard area of 50,000 pixel, which corresponds to a circular area of 2.63° radius. An example is shown in the center panel of Fig. 1. The standard and the test stimuli were presented for 0.5 s each in random order (2AFC). Between the two intervals a gray blank screen with a central fixation cross was shown for 0.75 s. After each stimulus pair a key press was awaited while only the fixation cross was presented. The test and standard positions were separately offset from the fixation point to avoid interference by afterimages and to prevent the observer from using landmarks on the screen for size judgments. The offset was randomly selected in both horizontal and vertical direction from a uniform distribution with a width of 75 arcmin (60 pixel). The test stimulus either varied in texture size with dot density kept constant at the level of the standard (left panel of Fig. 1) or in dot density with size kept constant at 2.63 arcmin radius (right panel). The number of dots co-varied with size or density, respectively. The deviation in either texture size or density relative to the standard patch was chosen by an adaptive procedure (Watt & Andrews, 1981) in steps of 4%. The procedure was designed to obtain the 50% point (*μ*) and the standard deviation (*σ*) of the psychometric function efficiently by concentrating cue values at *μ* ± *σ*.

Similar to the experiment with circular structures described in Raphael et al. (2012) the following conditions and Trial Types were used. We use ‘Condition’ to refer to a block of trials containing the same Task and one or two Trial Types and, ‘Trial Type’ to refer to the kinds of trial within a block.

The ‘Density Condition’ consisted of blocked density varying trials where the area of the test was the same as the standard and the density of dots co-varied with the number. Observers estimated the differences in density between the test and standard patch. Similarly, the ‘Size Condition’ consisted of size varying trials where the density of the dots in the test was the same as in the standard, and the area was adjusted to accommodate the greater or smaller number of dots at that fixed density. Here, observers were asked to estimate the differences in texture area. In both conditions, size varying and density varying trials were presented in separate blocks and observers made a binary choice: ‘denser’/‘less dense’ and ‘larger’/‘smaller’. In a modified Size Condition, the ‘Outline Size Condition’ only the outline of the polygon shape was shown but no dots. Here, observers compared area size of the test stimulus with the area size of the standard.

In the ‘Mixed Task Condition’ and in the ‘Numerosity Condition’ trials of size and density varying cues were randomly interleaved. In the ‘Mixed Task Condition’ observers were asked which kind of difference (size or density) was present, and the direction of change. In the ‘Number Condition’ the observers had only two keys available, to indicate which stimulus had more dots (numerosity discrimination).

Since we cannot prevent observers in the density and size conditions using numerosity as a cue (because both signals co-vary with numerosity), we introduced a further condition to estimate size (‘Extended Size Condition’) and density (‘Extended Density Condition’) changes alone. Here, we introduced a trial-type for which the number of dots was kept constant at 64 dots in each stimulus with size and density of the test varying oppositely to each other. Hence, in the Extended Size Condition in half of the trials a larger stimulus coincided with less density and in the 50% of trials a larger stimulus coincided with higher numerosity, but constant density compared to the standard. The aim of this arrangement is to prevented observers from using numerosity as a reliable cue to estimate the density or size of the texture.

An overview of all conditions is given in Table 1.

Prior to the experiments the observers were shown examples of the stimuli and were told about the relationship between density, size and number of dots in the different conditions.

Each observer completed 5 sessions of all conditions; only of the Mixed Task Condition 7 sessions were done. In the Single Trial Type conditions (Size Task, Density Task and Outline Size condition) the sessions contained 128 trials summing up to 640 trials per observer and condition. In the Mixed Task, Extended Size and Density and Number Conditions two different Trial Types were interleaved summing to at least 1280 trials per observer and condition.

Five observers took part in the experiments; three with experience in psychophysical experiments (Observers 1–3 of age 34, 37 and 70) and two with no previous psychophysical experience (Observer 4 and 5 of age 19 and 39). The vision of subjects 1, 3, 4 and 5 was normal or corrected to normal. Subject 2 was a myope with −0.75D/−0.75D. Subjects 1–4 took also part in the numerosity experiments presented in Raphael, Dillenburger, and Morgan (2013) and had therefore some experience in numerosity, size, and density judgment. The experiments were carried out in accordance with The Code of Ethics of the World Medical Association (Declaration of Helsinki) and informed consent was obtained from the human participants.

### 2.2 Data analysis and modelling

Individual psychometric functions representing percent ‘larger’/‘denser’/‘more’ responses of each condition and Trial Type were fit, using the MATLAB ‘fminsearch’ procedure, by cumulative Gaussian functions with parameters *μ* (50% point) and spread *σ* (standard deviation). 95% confidence limits for the individual points on the psychometric functions and those for the fitted parameters of the psychometric functions were obtained by a bootstrapping procedure with 640 simulations. The best-fitting parameters of the psychometric functions and their confidence intervals for all subjects and tasks are given in Table 2, Table 4.

To see if there were any statistically significant effects of Trial Type a Chi-squared test based on likelihood ratios was used. A fit to the combined data over Trial Type using different values of *σ* for the two Types was compared to a fit using the same value of *σ*. To asses the differences in only the slope of the psychometric functions (*σ*), the PMFs were allowed to have different *μ*’s. The likelihoods and the derived chi-squared values of these pairwise comparisons are shown in Table 2. Twice the difference in log likelihoods between the two fits was assumed to be distributed as Chi-squared with 1° of freedom (Hoel, Port, & Stone, 1971). If the two-*σ* fit is significantly better than a one-*σ* fit we can conclude that the thresholds for the two trial-types (density or size changes with numerosity) are significantly different.

Signal detection models were fit to all the data of a single subject under all the experimental conditions. Details are given at the appropriate point in the paper.

## 3 Results

**Fig. 2:**
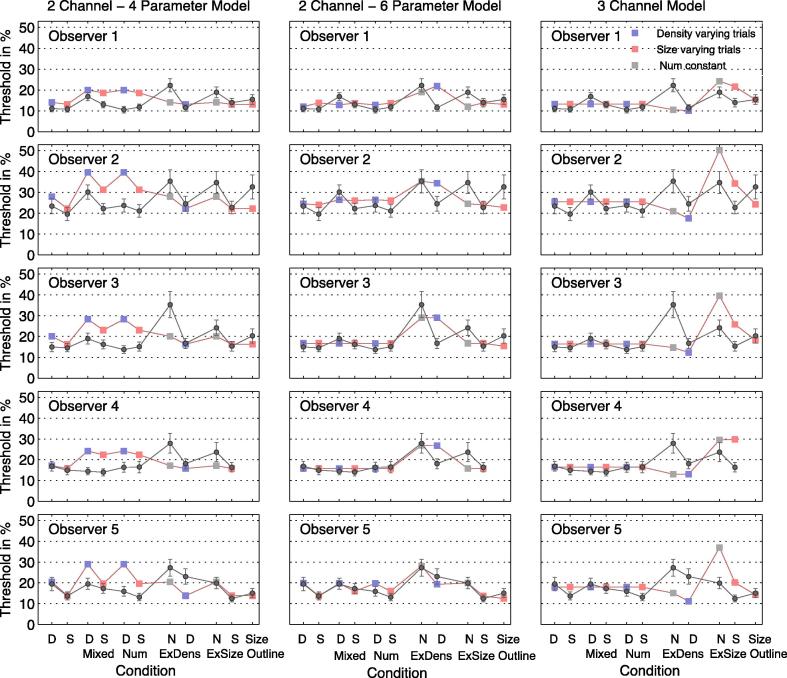
The three panels in each row compare psychophysical performance (circles) of one observer with the predictions of various models (square symbols). Each row is a different observer. Each symbol represents performance in one of the 11 conditions and Trial Types of the whole experiment. ‘D’ and ‘S’ refer to density and size varying Trial Types, respectively. The vertical axis shows the Weber fractions. The open circles are the observers’ thresholds of that condition; error bars are 95% confidence interval calculated with bootstrapping. The square symbols show the predictions of three different models (columns). The leftmost column shows thresholds of the 2 channel model with 4 parameters (*μ*_S_, *σ*_S_, *μ*_D_, *σ*_D_). The middle column shows predictions of the two channel model with extra parameters for leakage and choice bias (*μ*_S_, *σ*_S_, *μ*_D_, *σ*_D_, leakage, bias). The right hand column shows predictions of the model with separate channels for density, size and number (*μ*_S_, *σ*_S_, *μ*_D_, *σ*_D_, *μ*_N_, *σ*_N_). The fits of the models are constrained by the data of all conditions. This is why some of the fitted values are markedly discrepant with some data points. The symbols have been joined by lines solely for convenience of reading; in reality there is no continuity between the different conditions.

Thresholds (Weber Fractions) are shown separately for the different conditions, Trial Types and observers in Fig. 2 and Table 2. The observer’s thresholds (*σ*) are shown by circles with error bars indicating 95% confidence limits. The colored bars show predictions of a 2-channel size-density model, to be described below. The horizontal lines depict predictions of a 3-channel size-density-numerosity model.

For a given observer, thresholds are similar across tasks and conditions, and observers who are relatively good at one task are also good at the others. The most obvious feature of the data is that thresholds are similar for size, density and numerosity, both in Single Trial and mixed conditions (first 6 data points, reading from the left of the figure). Unlike the previously reported results for circular textures (Ross, 2010; Raphael, 2013) numerosity thresholds were not lower in the size-varying vs the density-varying conditions. The exception to this simple rule is in the ‘Extended Size’ and ‘Extended Density’ conditions. Here, thresholds are lower when size/density co-varies with numerosity than when size and density change in opposite directions, so as to keep number constant. Finally, thresholds are higher in the Outline Size condition, when only the outline of the polygon changes in size than in all the other conditions when dots are present within the outline.

These findings are confirmed by the statistical pairwise comparisons in Table 2.

**Fig. 3:**
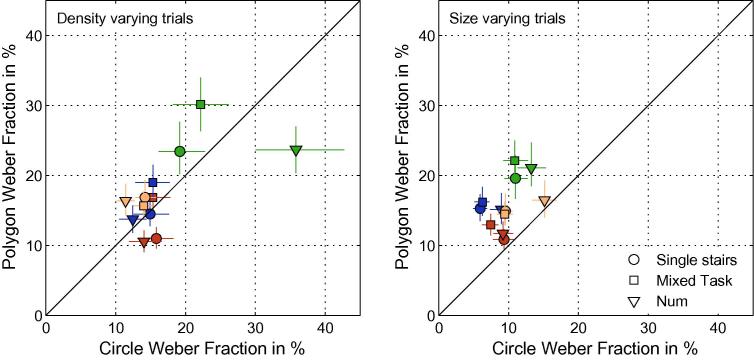
Thresholds (Weber Fractions) of the ‘Circles Experiment’ vs Thresholds of the ‘Polygons Experiment’, plotted separately for density varying trials (left panel) and size varying trials (right panel). Data for subjects 1–3 are indicated by symbol colors *Red*, *Green*, *Blue*. The shape of the symbols indicates the experimental condition. *Circles*: Single Trial Type size and density varying trials. *Squares*: size and density varying trials are randomly interleaved and the subject has to indicate not only in which direction the test stimulus is changed but also whether it differs in size or density (Mixed Task). *Downward pointing triangles*: size and density varying trials are randomly interleaved and the subject has to indicate whether the test has more or less dots than the standard (Numerosity Condition). Thresholds for size are raised in the Polygon experiment, but there is no systematic change for density. (For interpretation of the references to color in this figure legend, the reader is referred to the web version of this article.)

Fig. 3 and Table 3 compare the Weber fractions of the polygon experiment against those of the previously published circle experiment of the four subjects that took part in both experiments (Raphael, Dillenburger, & Morgan, 2013). It is shown that thresholds on size-varying trials are indeed lower with circular texture than for polygons in all conditions.

**Fig. 4:**
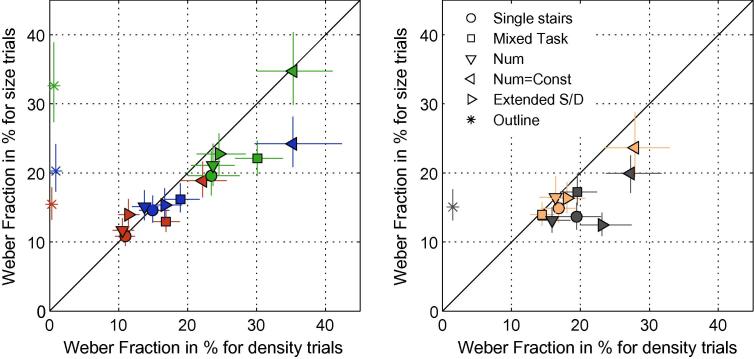
The figure plots thresholds on density varying trials against those on size varying trials. Data for subjects 1–3 are indicated by symbol colors *Red*, *Green*, *Blue* in the left panel and for subjects 4 and 5 in the right panel in *Orange*, and *Gray*, respectively. The shapes of the data points correspond to the experimental condition. Additionally, the *rightward pointing triangles* correspond to the trials of the Extended Size and Density Conditions where dot number co-varys with Size or Density, respectively. The *leftward pointing triangles show the thresholds for the* Trial Types of the Extended Size and Density Conditions with dot number kept constant. *The Stars* stand for the Outline Size Condition. The error bars represent 95% confidence limits. (For interpretation of the references to color in this figure legend, the reader is referred to the web version of this article.)

In Fig. 4 which shows the Weber Fractions of size varying trials against density varying trials for each observer and condition it can be seen that the thresholds for size-varying and density-varying trials, in size, density and number conditions are indeed similar. In trials of the Extended Conditions when numerosity is kept constant and density and size vary reciprocally, discrimination is significantly impaired (significant in 9 out of 10 comparisons, 5 observers× 2 Trial Types). Though, comparing the Single Trial Type conditions for size and density with the same Trial Type of the extended conditions of size and density reveals no increase in threshold for size judgment. Hence, observers are not worse in density and size judgments when stimuli are interleaved with trials that offer a less reliable cue.

When only the outlines of the polygons but no dots were shown the threshold for Size judgment increases markedly (significantly in 3 out of 4 observers) relative to the case where changes in size co-vary with dot number. The Weber Fractions in the outline Size Condition resemble the thresholds of the Extended Size Condition when numerosity is kept constant and does not co-vary with patch size. This suggests that the outline condition gives a true measure of size discrimination, when it is not aided by concomitant changes in density and/or number. Size discrimination of polygons thus appears to be relatively poor, as would be expected from the finding that the most accurate forms of 2-D size discrimination are obtained by combining 1-D estimates (Morgan, 2005).

**Fig. 5:**
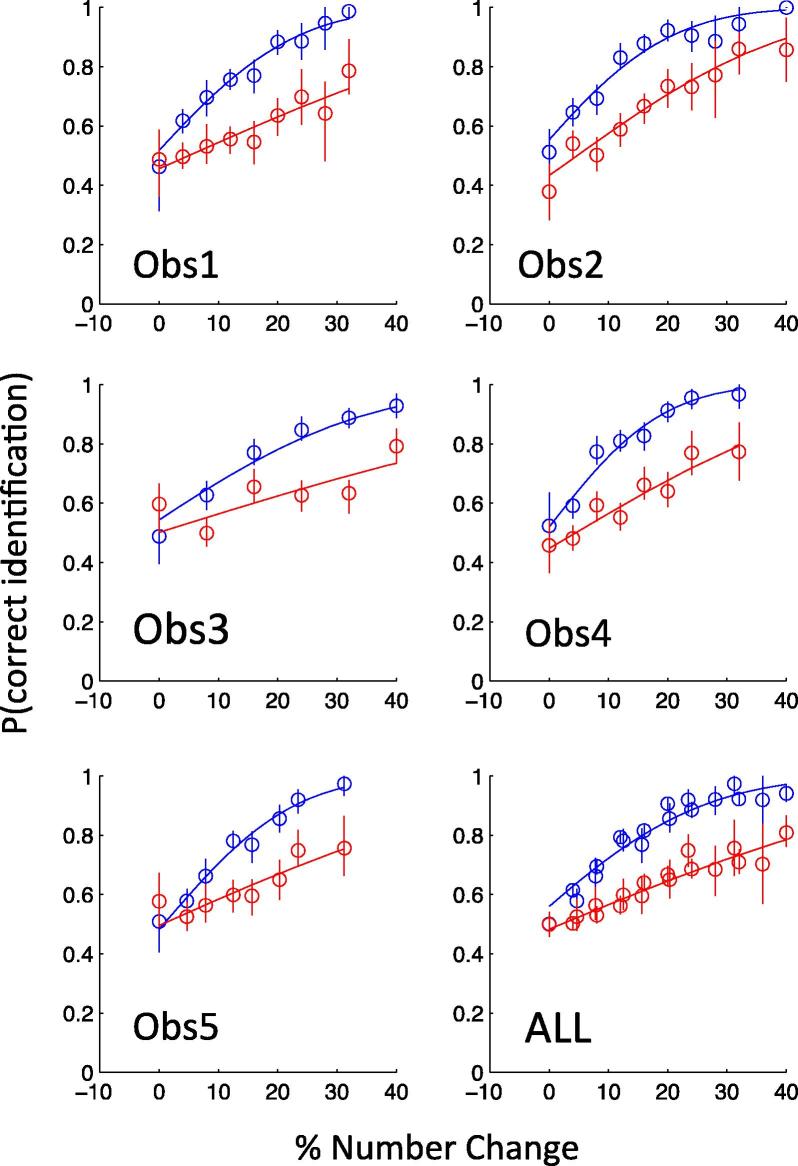
The figure shows that subjects were able to discriminate (Red) changes of size from changes in density when they were randomly interleaved (Mixed condition). Also shown (Blue) is the ability to discriminate the direction of the numerosity change (increase vs decrease). The first five panels show the data for each of the observers separately and the bottom right panel shows all the data combined over subjects. Error bars show 95% confidence limits based on the binomial distribution. The continuous curves are the best-fitting cumulative Gaussians. (For interpretation of the references to color in this figure legend, the reader is referred to the web version of this article.)

The simplest explanation of the identity of thresholds between size- density- and number-varying conditions is that the same mechanism is being used in each case: a relative numerosity system. However, a single-channel model for all the data is ruled out by a number of facts. One is that observers were able to discriminate changes in size and density in the Mixed Task Condition (see Fig. 5). Performance in this 4 button task, as measured from the slope of the psychometric functions, was not as good as that for discriminating the direction of the numerosity change (increase vs decrease) but this is to be expected from a 2-channel model where there are separate channels for size and density, as we show in the Modelling section below. Another problem for a single-channel numerosity model is that observers could still discriminate changes in size/density in the Extended Condition where numerosity was held constant, albeit with decreased accuracy versus the condition where numerosity changed also. Finally, observers could discriminate changes in size of an outline figure, where the numerosity signal was absent, albeit with reduced sensitivity.

These considerations suggest that a hybrid model may be required to explain the full range of data, incorporating size, density and numerosity mechanisms (channels). But if this approach is to be taken, it is necessary to show that a 3-channel model is significantly better than a 2-channel (size/density) model, taking into account the greater number of parameters in the 3-channel case. We address this question rigorously in the following Modelling section.

## 4 Modelling

### 4.1 Two-versus three-channel models

**Fig. 6:**
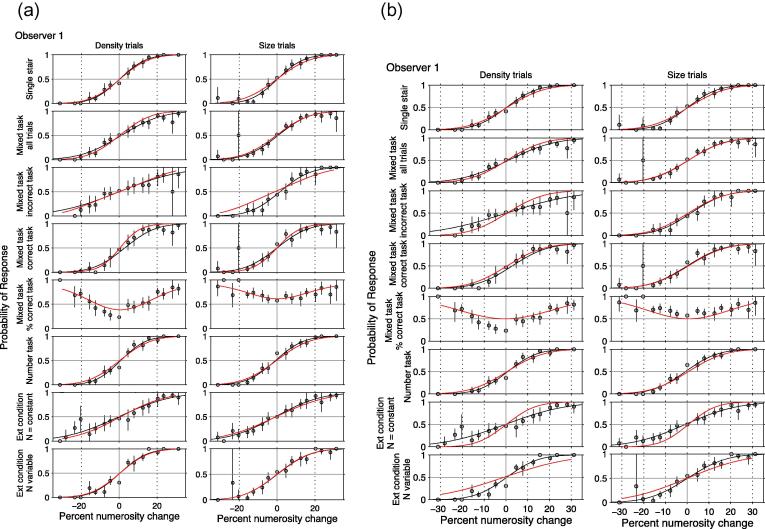
The figure shows the psychometric functions of one observer for different conditions and Trial Types and the fits of two different models. The left-hand figure (a) shows the fit of a 2-channel (Size and Density) model. The right-hand figure (b) shows the fit of a three-channel (Size, Density and Number) model. The vertical axis of the first 4 panel rows within each set show the probability of the response ‘denser’ (left column)/‘larger’ (right column). The *y*-axis of the bottom most panels depicts the probability of response ‘more’. Within each set the top two panels show the psychometric functions of density varying trials in the Density Task (left) and size varying trials (right) in the Size Task. The panels in rows 2–5 show the results on the Mixed Task when the size and density varying trials were randomly interleaved and the observer chose between 4 responses. Row 2 shows all trials under that condition. Row 3 shows the results when the observer chose the wrong task, e.g. responded to density when the patches had a size difference. Row 4 shows results on trials when the observer chose the correct task. Row 5 shows the probability of correctly choosing the density task on density trials (left) and correctly choosing the size task on size trials (right). Row 6 shows the results of the Numerosity Task, when the density and size trials were randomly interleaved and the observer chose between 2 responses (‘more’ vs. ‘less’). The error bars show 95% confidence levels. The solid black curves depict 2-parameter (*μ*, *σ*) fits to the individual psychometric functions (see Table 1). The red curves in the left-hand figure (a) show the best fits to all the data of the 2-channel 6-parameter MAX model with one parameter accounting for the bias towards selecting the density response over the size response. The blue curves in the right-hand figure show the fit of a 6-parameter model with 3 channels. The fitted parameter values for all observers are shown in Table 2.

We consider whether a numerosity channel is warranted by the data, or whether independent size and density channels will suffice. We note that in all conditions where numerosity was varied, size or density was varied as well: therefore a two-channel model deserves consideration on grounds of parsimony. To model the data we used the data from all Trial Types and Conditions including the Extended Conditions and the Outline Size condition. An example of the full data set for one observer and the fit of two models are shown in Fig. 6. The modelling results for all observers are shown in Fig. 2. In what we shall call the ‘2 channel model’ we assumed two independent mechanisms or channels, one responsive to size changes and the other to density. In Single Trial Type conditions the observer monitors only the relevant channel. Otherwise the observers are assumed to monitor two different noisy signals for size and density each defined by the shift (*μ*) and the sigma (*σ*) of the psychometric function (*μ*_S_, *σ*_S_, *μ*_D_, *σ*_D_) and chooses the one that deviates most from its reference value (Green and Swets, 1966, Palmer et al., 1993, Palmer et al., 2000; Morgan and Solomon, 2006). Having chosen the channel according to the Signal Detection Theory ‘Max’ rule, they choose their response on the basis of the sign of the deviation. The signals between which the observer chooses are assumed to be normalized by their standard deviation. This is particularly important, when the two channels have different noise levels since the observer would have strongly biased towards choosing the more noisy density signal (Raphael, Dillenburger, & Morgan, 2013).

To model the data in the extended size and Density Conditions, we assumed that observers monitored only the relevant channel (size or density) as in the Single Trial Type condition. In the size-outline condition, the observer monitors only the size channel.

To improve the fit of the two-channel model and to compensate for two conspicuous failures, two further parameters were included (as in Raphael, Dillenburger, & Morgan, 2013). The first discrepancy occurs because the channels are clearly not independent. On trials when the observer makes an incorrect identification of size vs density, they are above chance at reporting the correct direction of the change. In other words, an increase in density is more likely to be reported as an increase in size than as a decrease in size (see row 3 in Fig. 6). A correlation between observed size and density has previously been reported by (Dakin et al., 2011) who note precedents in the previous literature on density discrimination. The correlation takes the form of larger stimuli appearing denser, and denser stimuli appearing larger, the same as the correlation observed in the present data. To account for the cross talk between channels we introduced a ‘leakage’ parameter: a fixed proportion of the signal in the channel containing the signal was added to the channel not containing the signal. For example, if the signal on a particular trial were *m* in the density channel, a signal *mp* (*p *< 1) would be added to the size channel. This is equivalent to introducing a bias *μ* in the psychometric function. If *p *> 0 this ensures that observers will be above chance at detecting the direction of the numerosity difference even if they incorrectly identify its source. The same cross talk will also improve performance in the numerosity condition where observers do not have to identify the source. The cross-talk was also assumed to be present in the extended conditions, leading to a larger signal in the case where size and density co-varied and to a smaller signal when they counter-varied. There was no cross-talk in the outline size condition, because the density cue was absent.

A second major drawback of the simple 2-channel model is that it assumes observers choose density or size equally often if there is no noticeable cue available, hence there is no bias in the identification of the source of change. However, some observers (observers 1, 2, and 3) show a significant bias as can be seen in the upshift or downshift of the response probability for small cues in the 5th row of Fig. 6. Hence, a parameter that accounts for the bias for choosing density or size as the source was introduced, as explained in Raphael, Dillenburger, & Morgan, 2013;. The fitted values of this parameter showed large biases in Observers 1–3 (−0.29, −0.35 and −0.23, respectively) but no significant biases in Observers 4 and 5 (−0.01 and −0.02, respectively).

Next, we consider a 3-channel model with independent channels for size, density and numerosity. We fitted the same 2 parameter (*μ*, *σ*) psychometric function to all empirical functions where numerosity varied and could be used as a cue. However, a single mechanism could not discriminate between size and density changes in the Mixed Task, so we added two further channels for Size and Density, each with its own *μ* and *σ*, and deemed that observers used the Max rule to make their choice. This gave a 6-parameter model in total. The size and density channels are used only to identify the source of the numerosity difference in the Mixed Task condition and in the Extended Conditions. More details of the models are given in (Raphael, Dillenburger, & Morgan, 2013).

The fitted parameter values of the described models are shown in Table 2. For all observers, the 3-channel model was inferior to the 2-channel 6-parameter model. Predicted thresholds of the 3-channel model are overestimated in the extended conditions when numerosity is constant, and underestimated when numerosity co-varies with density or size. No significance levels can be assigned to these differences because the models are equally constrained, but the conservative conclusion is that we cannot reject the model of a single numerosity mechanism, which the observers use to make discriminations of ‘greater’ vs ‘smaller’ in all of the conditions, number, size and density. It is noteworthy that in the same analysis carried out for the circle experiment, a single number channel model fails badly in all observers, reinforcing the conclusion that there are at least 2 channels, for size and density. The difference between the two experiments is that observers are more sensitive to size of the circles than to the size of polygons.

It might reasonably be objected that the 3-channel model has been disadvantaged relative to the 2-channel model by having three values for *μ* instead of two. There is no reason to expect non-zero values for this bias parameter, and the fitted values are small. Therefore, we repeated the fits of the models with *μ* values constrained to be zero. The 6-parameter three-channel model now had only three parameters (*σ* for each channel) while the two-channel model with leakage and bias had four. The different degrees of freedom allow a statistical comparison between the fits (Hoel, Port, & Stone, 1971). The results of the fits are shown in Table 4. For every subject, the fit of the 2-channel model was superior to that of the three-channel model (see Table 5).

## 5 Discussion

It is clear from these data that the mechanisms for size, density and numerosity discriminations are closely intertwined. For a given observer, thresholds are similar across tasks and conditions, and observers who are relatively good at one task are also good at the others (Kendall’s Coefficient of Concordance = 0.75). Moreover, changes in density tend to be confused with changes in size. Whether these findings support the idea of a special numerosity mechanism, distinct from size and density, is a complex question, which we now address.

The main argument against a pure numerosity mechanism is that it cannot explain the ability of observers to discriminate between changes in size and density at threshold. Separate channels for size and density are required. Once these have been admitted, the introduction of a third channel for numerosity is difficult to justify. An augmented two-channel model to take account of cross-talk between channels, and allowing for biases in identification, has no more parameters (6) than the 3-channel model and is more successful. If the mean (*μ*) of the psychometric function is constrained to be zero, the resulting 3 parameter version of the three-channel model is significantly inferior to the 4-parameter version of the two-channel model for all observers. Further, the ability of observers to report the direction of a numerosity change correctly when they are unable to identify its origin (size vs. density) can be explained by a bias towards seeing denser patterns as larger and vice versa. This bias has been independently confirmed (Dakin et al., 2011) and could be explained by constancy scaling (Raphael et al., 2013, Thouless, 1972) or decorrelation of naturally-correlated signals (Barlow & Földiák, 1989). The bias can be modeled by a cross talk between the size and density channels. Note that the average fitted parameter value for this cross-talk over observers is 0.36, which is far less than the value of unity predicted from using a pure numerosity channel. As mentioned earlier, the increase in density thresholds when size changes in the opposite direction (constant number trial type in the Extended Density Condition) and vice versa can be explained by the cross-talk between size and density channels.

Our view is that the case for a distinct numerosity mechanism is ‘not proven’ by the present experiment. It cannot be rejected, nor is there any compelling reason to accept it. Lacking so far is a model for how numerosity is computed if it is indeed true that ‘Vision senses number directly’ (Ross & Burr, 2010). If counting is excluded for numbers outside the ‘subitizing region’ (Jevons, 1871, Ross and Burr, 2010) it is a simple matter of logic that approximate numerosity must be computed from some summary statistic of the image, such as contrast energy (Dakin et al., 2011, Morgan et al., 2014). However, Anobile, Cicchini, and Burr (2014) have recently suggested that mechanisms for numerosity and texture density are separable, the former operating only for numbers too small to constitute a texture. Their evidence is that Weber’s Law for numerosity is replaced by a square-root relation for large numbers. The challenge now is to provide a mechanism for approximate numerosity, other than a texture based mechanism, that explains the Weber relationship.

## Figures and Tables

**Table 1:** Summary of all conditions and Trial Types.

Condition	Trial Type 1	Trial Type 2
Density Condition ○	Density co-varies with numerosity, size const.	–
Size Condition ○	Size co-varies with numerosity, density const.	–
Mixed Task Condition □	Density co-varies with numerosity, size const.	Size co-varies with numerosity, density const.
Numerosity Condition ▽	Density co-varies with numerosity, size const.	Size co-varies with numerosity, density const.
Outline Size Condition *	Size co-varies with numerosity, density const.	–
Extended Size Condition ◁/▷	Size and density vary oppositely to each other, numerosity const.	Size co-varies with numerosity, density const.
Extended Density Condition ◁/▷	Size and density vary oppositely to each other, numerosity const.	Density co-varies with numerosity, size const.

**Table 2:** The table shows fitted values of the mean (*μ*) and standard deviation (*σ*) of the psychometric functions obtained under the conditions shown in the column headings. Also shown are the log likelihoods (*L*) of the separate fits (3rd row for each subject) and the Weber Fraction and the log likelihood of the combined size and density varying trials fit (4th row). The last row of each subject shows the *χ*^2^ of the likelihood ratio test for the difference between the individual fits for Size and Density and their combined fit and its significance level (^*^p < 0.05, ^**^p < 0.01, ^***^p < 0.001). The last column of the Single Trial Type conditions shows the *χ*^2^ values for the likelihood ratio test between size judgments with dots and with the polygon outline.

		Single Trial Type			Mixed Task		Numerosity Task		Extended Dens		Extended Size	
Dens	Size	Outline	Dens	Size	Dens	Size	N const.	Dens	N const.	Size
1	*μ*	0.25	0.23		1.19	0.54	0.81	−0.84	0.28	0.41	−0.91	0.17
*σ*	10.99	10.85	15.46	16.84	12.95	10.56	11.7	22.21	11.4	18.87	13.94
*L*	287.21	270.77		437.05	395.05	290.92	293.34	335.67	291.12	312.42	317.46
	10.92		12.63^∗∗^	14.95		11.14		17.17		16.66	
*χ*^2^	0.02		9.34^∗∗^		1.11		39.86^∗∗∗^		8.40^∗^	

2	*μ*	−1.17	1.49	32.60	5.79	0.16	4.33	0.27	2.55	−1.42	4.19	2.18
*σ*	23.42	19.58	30.11	22.12	23.68	21.08	35.3	24.55	34.74	22.76
*L*	353.70	349.73	419.34	379.19	280.44	286.27	335.47	310.26	345.52	283.81
	21.57		16.84^∗∗∗^	26.17		22.47		30.10		28.67	
*χ*^2^	2.35		13.75^∗∗^		1.41		11.15^∗∗^		16.27^∗∗∗^	

3	*μ*	0.77	1.13	20.27	1.54	0.92	−0.85	0.13	1.72	−0.22	3.79	0.73
*σ*	14.92	14.60	18.97	16.20	13.75	15.13	35.26	16.68	24.21	15.34
*L*	319.72	308.70	468.94	471.68	304	313.69	367.21	325.49	315.03	333.68
	14.75		8.59^∗^	17.62		14.44		26.64		20.49	
*χ*^2^	0.05		2.87		0.9		39.39^∗∗∗^		16.99^∗∗∗^	

4	*μ*	−0.58	0.43	–	−0.48	0.64	2.21	1.30	2.56	−0.98	1.80	0.10
*σ*	16.85	14.90	14.406	13.94	16.38	16.46	27.91	18.05	23.66	16.34
*L*	305.86	330.21	436.66	431.46	324.31	326	348.87	310.49	368.59	304.85
	15.98			14.17		16.42		22.98		19.6	
*χ*^2^	1.38		0.15		0.00		16.06^∗∗∗^		10.73^∗∗^	

5	*μ*	1.39	1.72	15.06	0.87	0.01	3.00	−0.20 1.3278	−0.35	0.25	1.04	−0.47
*σ*	19.43	13.65	19.54	17.25	15.90	13.15	27.26	23.11	19.92	12.46
*L*	348.17	302.37	484.84	468.02	334.07	304.34	326.5	350.3	338.6	295.0
*σ*	16.42		0.95	18.4		14.5		25.45		16.22	
*χ*^2^	11.17^∗∗^		1.76		3.4		2.23		20.26^∗∗∗^	

**Table 3:** The table shows the mean (*μ*) and standard deviation (*σ*) of the fitted c functions and the log likelihood (*L*) of the fits. Also shown in the column labeled Mean/*L* are the values of *μ* and *σ* for the combined fits of the circle and polygon data and the associated log likelihood. The column headed *χ*^2^ shows the results of the likelihood ratio test for the difference between the individual fits for circles and polygons and their combined fit and the rightmost column show the significance for df = 1.

Obs	Task	Circle			Polygon			Combined			*χ*^2^	Sign
*μ*	*σ*	*L*	*μ*	*σ*	*L*	*μ*	*σ*	*L*
1	Dens Task	3.82	15.81	242.66	0.25	10.99	287.21	2.01	13.12	535.67	11.59	**
Size Task	2.13	9.35	182.44	0.23	10.84	270.77	1.22	10.20	454.24	2.05	–
Mixed – Dens	−0.33	15.30	225.77	1.19	16.84	437.05	0.43	16.29	663.26	0.87	–
Mixed – Size	0.18	7.45	154.30	0.54	12.95	395.05	0.25	11.10	563.74	28.79	***
Num – Dens	−1.48	14.03	302.30	0.81	10.56	290.92	−0.21	12.09	596.77	7.11	*
Num – Size	−0.86	9.19	255.28	−0.84	11.70	293.34	−0.87	10.59	551.43	5.62	–

2	Dens Task	0.16	19.17	257.65	−1.17	23.42	353.70	−0.55	21.48	612.75	2.79	–
Size Task	2.65	10.97	192.77	1.49	19.58	349.73	2.25	15.53	556.09	27.18	***
Mixed – Dens	2.96	22.16	277.41	5.79	30.11	419.34	4.20	28.01	700.26	7.02	*
Mixed – Size	1.90	10.89	202.06	0.16	22.12	379.19	1.00	18.76	603.43	44.36	***
Num – Dens	2.47	35.82	280.38	4.33	23.68	280.44	3.27	28.58	567.83	14.03	***
Num – Size	1.49	13.23	239.73	0.27	21.08	286.27	1.05	18.03	534.88	17.76	***

3	Dens Task	4.30	14.91	246.69	0.42	14.48	379.16	2.36	14.64	625.88	0.07	–
Size Task	−0.81	5.93	160.44	0.88	15.27	381.13	0.09	12.13	576.01	68.88	***
Mixed – Dens	6.18	15.32	243.58	1.54	18.97	468.94	3.93	17.61	714.52	4.00	–
Mixed – Size	2.76	6.27	144.33	0.92	16.20	471.68	2.07	12.83	652.99	73.97	***
Num – Dens	4.93	12.43	205.70	−0.85	13.75	304.00	2.09	13.16	510.17	0.93	–
Num – Size	2.90	8.95	178.59	0.13	15.12	313.69	1.64	12.50	504.41	24.26	***

4	Dens Task	−2.41	14.19	421.32	−0.58	16.85	305.86	−1.47	15.21	729.08	3.81	–
Size Task	−4.78	9.52	354.45	0.43	14.9	330.21	−2.10	11.53	697.34	25.36	***
Mixed – Dens	0.34	14.00	398.21	−1.02	15.64	512.96	−0.32	14.83	912.08	1.80	–
Mixed – Size	0.46	9.45	313.75	0.64	14.47	506.30	0.62	12.08	834.09	28.07	***
Num – Dens	−2.34	11.41	387.55	2.21	16.38	324.31	−0.21	13.31	720.20	16.69	***
Num – Size	−2.74	15.15	450.80	1.30	16.46	326.00	−0.66	15.62	777.21	0.80	–

**Table 4:** The table shows best-fitting parameter values for 3 models described in the text and tests of significance between the models. The first model is the 5 parameter MAX model with 2 channels (*μ*_S_, *σ*_S_, *μ*_D_, *σ*_D_) and a leakage factor. The second line shows the 2 channel 6 parameter model which is supplemented by a bias in the choice between size and density channels. The third model has 3 separate channels for number, density and size. The columns 1 denote the observer. The last column contains the results of a likelihood ratio comparison between two models indicated in the previous column. For each observer the values of *χ*^2^ and the significance levels are shown.

Obs	Model	*μ*_D_	*σ*_D_	*μ*_S_	*σ*_S_	Leakage or *μ*_N_	Bias or *σ*_N_	*L*		*χ*^2^
1	2 Ch – 5 Param	0.43	12.47	0.10	12.10	0.36		4716.866		
2 Ch – 6 Param	0.39	11.03	−0.02	13.19	0.37	−0.29	4682.302	5 P vs 6 P	69.1^∗∗∗^
3 Ch	−0.26	17.13	0.60	15.28	0.51	13.28	4722.129	5 P vs 3 Ch	10.5^∗∗^

2	2 Ch – 5 Param	0.41	26.60	1.13	20.45	0.30		4877.382		
2 Ch – 6 Param	0.34	23.55	0.89	22.79	0.31	−0.35	4828.647	5 P vs 6 P	97.5^∗∗∗^
3 Ch	−1.98	35.58	4.94	24.28	1.27	25.47	4870.829	5 P vs 3 Ch	13.1^∗∗^

3	2 Ch – 5 Param	0.12	16.85	0.89	14.28	0.41		5032.732		
2 Ch – 6 Param	0.10	15.38	0.78	15.42	0.42	−0.23	5008.309	5 P vs 6 P	48.8^∗∗∗^
3 Ch	−1.17	27.97	2.17	18.30	0.49	16.48	5020.812	5 P vs 3 Ch	23.8^∗∗∗^

4	2 Ch – 5 Param	−0.89	14.65	−0.12	14.46	0.41		4689.785		
2 Ch – 6 Param	−0.89	14.60	−0.12	14.51	0.41	−0.01	4689.759	5 P vs 6 P	0.05
3 Ch	−2.99	20.90	0.79	21.07	0.34	14.49	4711.368	5 P vs 3 Ch	42.2^∗∗∗^

5	2 Ch – 5 Param	0.85	19.47	0.29	12.47	0.29		5055.687		
2 Ch – 6 Param	0.84	19.56	0.29	12.41	0.29	0.02	5055.536	5 P vs 6 P	0.03
3 Ch	2.06	26.14	-0.18	14.29	0.68	17.97	5084.682	5 P vs 3 Ch	58.0^∗∗∗^

**Table 5:** Best fitting parameters of the models with *μ* values constrained to be zero and the likelihood (*L*) for each observer. The *χ*^2^ values of the 6th and 12th column depict the results of a likelihood comparison test between the full models and the model with *μ* set to 0. The last column shows the *χ*^2^ value of the likelihood ratio test comparing the two constraint models.

Obs	3 Channel Model					2 Channel Model						Comparison of the models
*σ*(N)	*σ*(D)	*σ*(S)	*L*	*χ*^2^	*σ*(D)	*σ*(S)	Leak	Bias	*L*	*χ*^2^	*χ*^2^
1	13.35	17.06	15.38	**4724.4**	4.66	11.08	13.17	0.37	−0.29	**4683.4**	2.22	**82.1^∗∗∗^**
2	25.72	35.77	25.33	**4886.5**	31.5^∗∗∗^	23.52	22.94	0.31	−0.35	**4829.9**	2.55	**113.3^∗∗∗^**
3	16.57	27.73	18.78	**5026.1**	10.65^∗^	15.30	15.58	0.43	−0.23	**5010.6**	4.62	**31.1^∗∗∗^**
4	16.57	20.62	21.37	**4716.7**	10.80^∗^	14.49	14.61	0.41	−0.01	**4692.8**	6.00^∗^	**48.0^∗∗∗^**
5	18.14	26.45	14.24	**5087.7**	6.23	19.75	12.37	0.29	0.02	**5057.1**	3.12	**61.4^∗∗∗^**
